# Real-World Experience With Dapagliflozin in Non-diabetic Chronic Kidney Disease: A Case Series From India

**DOI:** 10.7759/cureus.107542

**Published:** 2026-04-22

**Authors:** Atanu Pal, Sourav Sadhukhan

**Affiliations:** 1 Rheumatology, ILS Hospital, Kolkata, IND; 2 Nephrology, Institute of Post Graduate Medical Education & Research, Kolkata, IND

**Keywords:** chronic kidney disease, dapagliflozin, iga nephropathy, non-diabetic ckd, proteinuria, sglt2 inhibitors

## Abstract

Introduction: Sodium-glucose cotransporter-2 inhibitors (SGLT2is), including dapagliflozin, have demonstrated reno-protective effects independent of glycemic control in chronic kidney disease (CKD). The data presented here add to the already available evidence on the use of dapagliflozin in patients with non-diabetic kidney disease

Methods: Non-diabetic CKD patients treated with dapagliflozin at a tertiary care center in India were included in the case series. Data for parameters, including renal function (estimated glomerular filtration rate (eGFR) and serum creatinine), proteinuria, and metabolic parameters, were retrieved retrospectively.

Results: Data of six non-diabetic patients with glomerular disorders (five with IgA nephropathy, one with membranous nephropathy) treated at a tertiary care center were collected. An initial decline in eGFR was observed at two weeks post-initiation of dapagliflozin, followed by significant recovery at six months (Day 14: 38.83 ± 9.21 ml/min/1.73 m² vs. Day 180: 44.83 ± 9.78 ml/min/1.73 m²; *P* = 0.0052). Proteinuria demonstrated progressive reduction across a six-month duration. Lipid profile analysis indicated a reduction in triglycerides but no significant changes in total cholesterol. No adverse events were reported.

Conclusion: Dapagliflozin was well tolerated and associated with improvements in renal function and proteinuria in non-diabetic CKD patients with autoimmune glomerular disorders. These findings support the advantages of dapagliflozin treatment in reducing the progression of CKD in non-diabetic patients.

## Introduction

Chronic kidney disease (CKD) is recognized as a gradually progressing condition that affects more than 800 million people worldwide and has emerged as one of the major causes of mortality [1]. CKD was responsible for 44.5 million disability-adjusted life years in 2021, and this includes those with end-stage renal disease (ESRD) and those who were on dialysis or had a transplant [1]. In India, the prevalence of CKD is about 16% (95% CI 12-21%) [2].

Considering the limitations in renal replacement therapy and the associated financial burden, there is increasing emphasis on early screening and management of underlying risk factors in patients with CKD, including both diabetic and non-diabetic etiologies. The newer therapeutic agents, such as sodium-glucose cotransporter-2 inhibitors (SGLT2is), have been shown to have substantial benefit in reducing the progression of CKD. The renal benefits of SGLT2i are mediated by natriuresis. SGLT2i primarily reduces sodium glucose reabsorption, thus restoring the tubule-glomerular feedback to a physiological state. In addition, SGLT2i is strongly associated with Na+-H+ exchanger 3 (NHE3), and suppression of NHE3 contributes to its natriuretic effect. Van Bommel et al. demonstrated that dapagliflozin treatment decreased glomerular filtration fraction initially without raising renal vascular resistance [3]. The data that have emerged with SGLT2i show that the post-glomerular vasodilatation impacts glomerular filtration. Therefore, the renal protective effects of SGLT2i may be termed as independent of effects on glucose or glomerular tubule feedback. SGLT2 increases distal sodium delivery and inhibits tubule-glomerular feedback, resulting in efferent vasoconstriction and reduction in intraglomerular pressure, and improving GFR. These mechanisms could preserve long-term kidney function [3-5]. 

Interestingly, animal studies have shown that the nephroprotective action of dapagliflozin is attributable to its induction of the renal expression of nephroprotective hypoxia-inducible factors. Most of these novel mechanisms are not shared by other reno-protective agents, such as angiotensin-converting enzyme (ACE) inhibitors or angiotensin II receptor blockers (ARBs). SGLT2is are distinct due to their ability to reduce energy consumption and hypoxia and their independent anti-inflammatory and antifibrotic effects.

In Dapagliflozin and Prevention of Adverse Outcomes in CKD (DAPA-CKD) trial, dapagliflozin was superior to placebo in reducing the risk of major adverse kidney outcomes and prolonging overall survival in a group of patients with albuminuric CKD [6]. The primary results indicated a 50% reduction in the risk of a composite of a sustained decline in the estimated glomerular filtration rate (eGFR) of at least 50%, end-stage kidney disease, or death from renal or cardiovascular causes (hazard ratio (HR): 0.50 (95% CI, 0.35 to 0.72)) in patients with CKD and normoglycemia compared to placebo [6]. The DAPA-CKD trial was conducted in CKD patients with or without type 2 diabetes. The observed reno-protective effects in patients with albuminuria and CKD without diabetes provide a rationale for the use of these agents as reno-protective therapies in patients with CKD due to causes other than diabetes [7].

Among the non-diabetic causes, IgA nephropathy is the most common primary glomerular disease, demonstrating declining eGFR, which may progress to kidney failure. IgA nephropathy and membranous nephropathy are autoimmune disorders that lead to excessive inflammation in the kidneys [8]. About 6.3% of patients in the DAPA-CKD trial had IgA nephropathy [6]. Immunosuppressive therapy remains a key component in the management of selected patients with primary glomerular diseases such as IgA nephropathy and membranous nephropathy [9]. However, patients included in clinical trials often have stringent inclusion criteria and may not fully represent real-world populations with CKD. Real-world evidence can therefore provide additional insights into the utility of SGLT2is, particularly dapagliflozin, in patients with non-diabetic CKD. This case series aims to describe real-world clinical experience and treatment patterns associated with dapagliflozin use in patients with non-diabetic CKD secondary to primary glomerular disorders.

## Materials and methods

This retrospective observational case series was conducted at a tertiary care nephrology unit in India between January 2022 and June 2023. The study involved a review of existing medical records to describe clinical outcomes of dapagliflozin in patients with non-diabetic CKD secondary to autoimmune glomerular disorders.

Study setting and patient identification

Patients were identified from the medical records of the tertiary hospital. All adult patients aged ≥18 years with a confirmed histopathological diagnosis of IgA nephropathy or membranous nephropathy, without type 2 diabetes mellitus, were identified. Eligible patients had an eGFR ≥30 mL/min/1.73 m² and were prescribed dapagliflozin 10 mg daily as an adjunct to standard therapy.

Inclusion and exclusion criteria

Inclusion criteria included patients with non-diabetic CKD, proteinuria between 500 mg and 5,000 mg/day, and an eGFR of 30-60 mL/min/1.73 m². Patients were required to be on a stable, maximally tolerated dose of a renin-angiotensin-aldosterone system (RAAS) blocker and not receiving any immunosuppressive therapy for at least six months prior to inclusion.

Exclusion criteria included patients with diabetes mellitus, acute kidney injury, active infection (including tuberculosis or HIV), malignancy, or pregnancy.

Intervention

All participants received dapagliflozin 10 mg once daily in addition to maximally tolerated doses of telmisartan (80 mg/day) or other renin-angiotensin system blockers. In patients with IgA nephropathy, corticosteroid therapy had been completed or discontinued prior to dapagliflozin initiation as part of routine clinical care. No new immunosuppressive agents were introduced during the study period.

Treatment approach and definitions

Patients with IgA nephropathy had received prolonged corticosteroid therapy as part of routine clinical care. Following discontinuation of corticosteroids, patients were observed for approximately six months to assess disease course.

Non-response to prior therapy was defined clinically as persistence or worsening of proteinuria, along with a rise in serum creatinine and/or a decline in eGFR during follow-up.

Dapagliflozin was initiated in patients with an eGFR between 30-60 mL/min/1.73 m² who demonstrated ongoing disease activity despite prior therapy. SGLT2i therapy was used as an adjunct to standard care and not as a replacement for immunosuppressive treatment.

The patient with membranous nephropathy had not received any immunosuppressive therapy at any stage of the disease, including corticosteroids, and was managed conservatively with renin-angiotensin system blockade alone. Dapagliflozin was added as an adjunctive renoprotective therapy.

All patients had CKD stage G3; however, further subclassification into G3a and G3b was not consistently available due to the retrospective nature of the study.

Measurements, duration, and outcomes

Data were collected for renal parameters (eGFR, serum creatinine, urine protein), serum albumin, lipid profile, and blood pressure. Renal function and biochemical parameters were assessed at baseline, day 14, day 60, day 120, and day 180 post-dapagliflozin initiation. The primary outcome measure included the change in eGFR. Secondary outcomes included changes in proteinuria, serum creatinine, triglycerides, total cholesterol, and safety events.

Data collection and handling

Patient-specific data were anonymized and recorded using a standardized data collection sheet. All laboratory parameters were extracted from patient medical records and validated by the investigator.

Statistical analysis

Given the small sample size, non-parametric tests were used to compare means over time. Descriptive statistics (mean ± SD) were used for continuous variables, and categorical variables were expressed as frequencies or percentages. Repeated-measures one-way ANOVA (Friedman test) was employed to evaluate temporal changes in eGFR, proteinuria, and lipid parameters. A p-value < 0.05 was considered statistically significant. Statistical analysis was performed using GraphPad Prism version 9.0 (GraphPad Software, La Jolla, CA, USA).

## Results

A total of six adult patients (three males and three females) with non-diabetic CKD are presented. At baseline (initiation of dapagliflozin), eGFR across patients ranged from 30-60 mL/min/1.73 m². Five patients were diagnosed with IgA nephropathy and one with membranous nephropathy. A summary of patient-wise demographic and clinical characteristics, along with blood pressure readings, is presented in Table 1.

**Table 1:** Clinical characteristics of the patients SBP: systolic blood pressure; DBP: diastolic blood pressure; IgAN: IgA nephropathy; MN: membranous nephropathy; CKD: chronic kidney disease; eGFR: estimated glomerular filtration rate

Case	1	2	3		4		5		6
Age (years)	49	43	42		36		43		26
Gender	Male	Female	Male		Female		Female		Male
Diagnosis	IgA N	IgA N	IgA N		IgA N		IgA N		MN
Weight (kg)	72	65	76		61		53		59
SBP (mmHg)	128	121	140		134		143		133
DBP (mmHg)	76	87	90		76		89		93
eGFR (mL/min/1.73m²)	49	33	56		33		46		56
Albumin (g/dL)	3.2	3.3	3.1		3.1		3.2		3.3
Urine proteins (g/Day)	2.1	1.76	2.16		1.61		1.92		1.75
Stage of CKD	3	3	3		3		3		3

**Figure 1:**
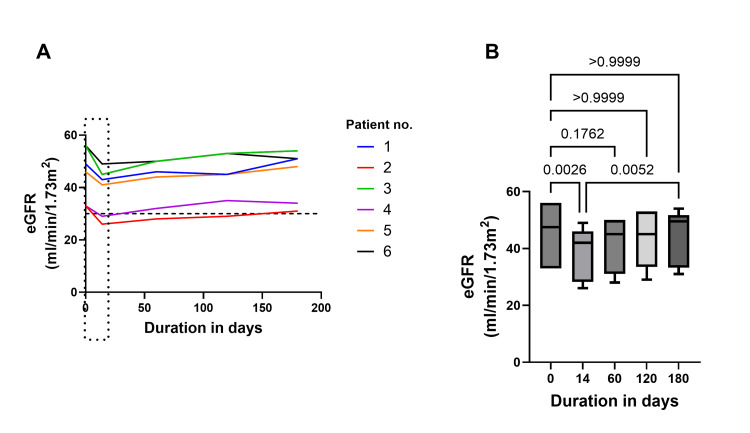
Trend in eGFR following treatment with dapagliflozin Figure 1A represents the patient-wise trend in eGFR up to 180 days. Lines of different colors correspond to individual patients. The dashed trend line indicates eGFR of 30 ml/min/1.73 m². The dotted box indicates the initial dip in eGFR following treatment with dapagliflozin. Figure 1B represents the distribution of eGFR values with 95% CI for a follow-up duration of up to 180 days. p-values added above the bars. eGFR: estimated glomerular filtration rate

**Figure 2:**
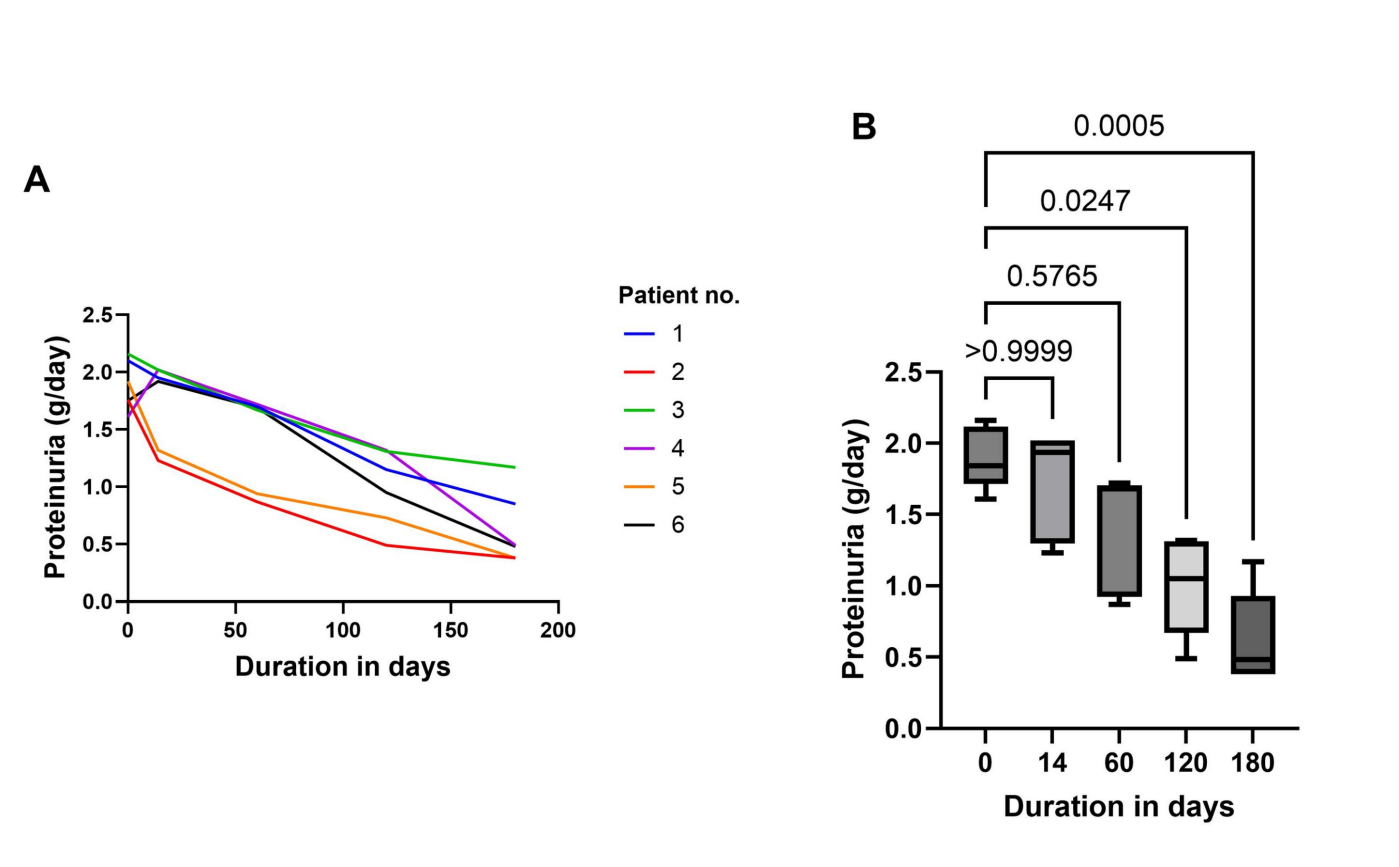
Trend in proteinuria following treatment with dapagliflozin Figure 2A represents the patient-wise trend of proteinuria levels up to 180 days. Lines of different colours correspond to individual patients. Figure 2B represents the distribution of proteinuria values with 95% CI for a follow-up duration of up to 180 days. The numbers above the bars indicate p-values.

There was a significant fall in eGFR at day 14 compared to baseline (45.5±10.5 ml/min/1.73m^2 ^to 38.8±9.2 ml/min/1.73m^2^; P=0.0026); however, eGFR significantly improved at day 180 compared to day 14 (38.8±9.2 ml/min/1.73m^2^ vs. 44.8±9.8 ml/min/1.73m^2^; P=0.0052; Figures 1A-1B). Following dapagliflozin treatment for six months, a significant decrease in proteinuria was observed at day 180 (Baseline 1.88 ± 0.22 g/day vs Day 180: 0.63 ± 0.32 g/day; P = 0.0002; Figure 2).

**Figure 3:**
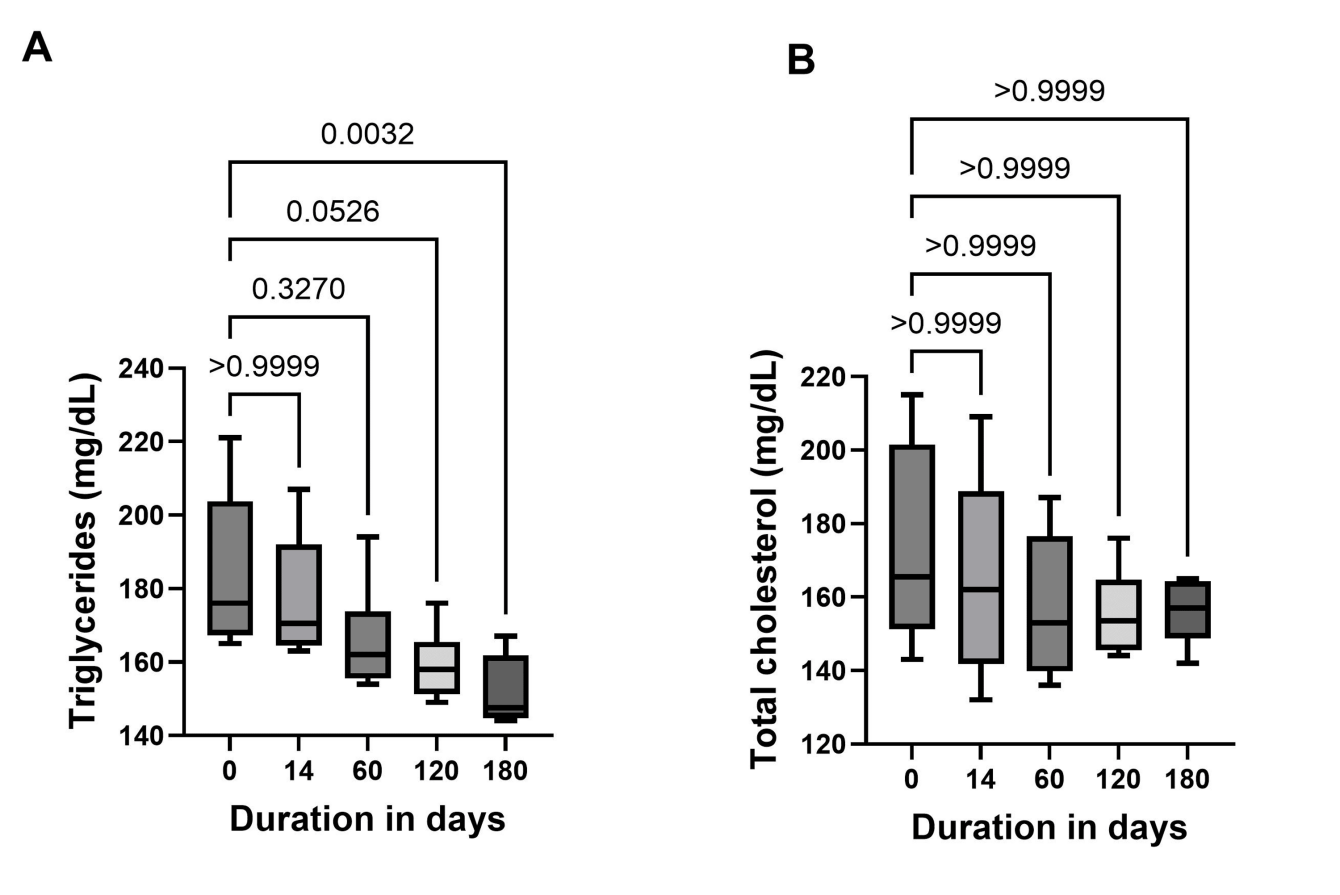
Lipid profile of the patient population following treatment with dapagliflozin Figure 3 represents the distribution of values with 95% CIs for clinical parameters: A) triglycerides, and B) total cholesterol for a duration of up to 180 days. The numbers above the bars indicate p-values.

Lipid profile evaluation demonstrated a significant reduction in mean triglyceride levels over the six-month follow-up period from baseline (Day 180: 184.0±21.73 mg/dL vs baseline: 159.2±9.81 mg/dL, p=0.0032) (Figure 3A), while total cholesterol values remained comparable to baseline during the same period (Figure 3B). 

Safety

No adverse events were reported during the study period. All patients had continued treatment with dapagliflozin.

## Discussion

This case series highlights the clinical utility of dapagliflozin in Indian patients with non-diabetic CKD. In this series, dapagliflozin was used as adjunctive therapy following standard clinical management rather than as a substitute for immunosuppression. The observed improvements in eGFR, reduction in proteinuria, and favorable safety profile are consistent with findings from larger trials of SGLT2is. These results add real-world evidence supporting the role of dapagliflozin in CKD management beyond diabetes.

Dapagliflozin, an antidiabetic agent from the class of SGLT2i, has proven cardiorenal benefits. The observations from large clinical trials such as DAPA-CKD and EMPA-KIDNEY (The Study of Heart and Kidney Protection With Empagliflozin) have demonstrated the potential of SGLT2i in managing CKD, even in the absence of diabetes [10, 6].

A subgroup analysis of the DAPA-CKD study has demonstrated the benefits of dapagliflozin in patients with IgA nephropathy. Dapagliflozin reduced the urinary albumin-to-creatinine ratio by 26% as against placebo [6]. Dapagliflozin significantly reduced the composite of sustained decline in eGFR of 50% or more, end-stage kidney disease, or death from kidney disease or cardiovascular causes in participants with CKD, including those with IgA nephropathy (hazard ratio, 0.29; 95% CI, 0.12, 0.73; P=0.005) [7].

A systematic review and meta-analyses demonstrated varying reductions in proteinuria: 76% over a 24-week period, 51% over four weeks, and 29% over three months with SGLT2i treatment [11-14]. Similarly, another study in a cohort of Chinese patients with IgA nephropathy on SGLT2i treatment observed a 22.9% and 27% reduction in proteinuria at three-month and six-month follow-up, respectively [15]. 

This case series presents a total of six clinical cases that demonstrate the real-world experience of dapagliflozin in the management of IgA nephropathy and membranous nephropathy. As anticipated, dapagliflozin initiation was associated with an early decline in eGFR, followed by recovery over the subsequent six months, which is an SGLT2i drug class effect (Figure 1). No adverse events occurred, and all patients had continued dapagliflozin therapy. Clinical trials and real-world studies have shown that initiation of SGLT2i leads to an initial dip in eGFR, followed by long-term improvement in renal function. Similarly, in this case series, there was an initial drop in eGFR levels within the first two weeks, followed by a positive slope that persisted over a six-month duration (Figure 1). In CKD patients, an increase in eGFR by 5 ml/min/1.73 m²/year is associated with significant improvement in kidney function [16]. The anti-inflammatory properties of dapagliflozin, combined with its mechanism of action and the associated significant reduction in proteinuria, support its role in renal protection and in slowing the progression of kidney disease to end-stage renal failure. SGLT2is demonstrate modest lipid-modifying benefits, typically reducing triglyceride levels and increasing high-density lipoprotein cholesterol (HDL-C) while exerting a variable impact on LDL-C levels [17]. There was a significant reduction in triglyceride levels (Figure 3). The real-world evidence of these clinical cases with IgA nephropathy and membranous nephropathy aligns with the clinical trial observations indicative of the therapeutic benefits of dapagliflozin in individuals with non-diabetic CKD.

Strengths and limitations

A key strength of this study is its real-world clinical context, providing insight into the use of dapagliflozin in patients with non-diabetic CKD secondary to primary glomerular disorders, a population that may be underrepresented in controlled clinical trials.

The small sample size and retrospective design limit generalizability and preclude causal inference; the findings should therefore be considered descriptive and hypothesis-generating. Treatment decisions were based on clinician judgment rather than standardized protocols. Baseline clinical data, including initial eGFR, proteinuria, and detailed immunosuppressive treatment parameters, were not consistently available. The duration of follow-up (six months) limits the assessment of long-term renal outcomes and safety. In addition, adverse event data were not systematically captured, and underreporting of rare or delayed effects cannot be excluded. PLA2R status and detailed immunological profiling were also not consistently available. Finally, the evolving therapeutic landscape in glomerular diseases such as IgA nephropathy may limit the contemporary applicability of these observations.

## Conclusions

This case series describes real-world clinical observations of dapagliflozin use in patients with non-diabetic CKD secondary to primary glomerular disorders. Improvements in proteinuria and stabilization of renal function were observed across cases, with a possible trend toward favorable changes in lipid parameters. These findings are consistent with previously published data; however, given the small sample size and retrospective design, they should be interpreted with caution. The observations are hypothesis-generating and highlight the potential role of dapagliflozin as part of a comprehensive renoprotective strategy in non-diabetic CKD.
